# A rare collision tumor of lymphoepithelioma-like carcinoma and t-cell lymphoma: a case report

**DOI:** 10.3389/fonc.2026.1855118

**Published:** 2026-05-29

**Authors:** Mengping Zhu, Junyu Zhang

**Affiliations:** Department of Hematology, Lishui Municipal Central Hospital, Lishui, Zhejiang, China

**Keywords:** collision tumor, lymphoepithelioma-like carcinoma, pathological diagnosis, T-cell lymphoma, case report

## Abstract

We report a rare case of a collision tumor in a patient concurrently diagnosed with lymphoepithelioma-like carcinoma (LELC) and T-cell non-Hodgkin lymphoma (NHL-T). The patient was a 61-year-old male presenting with a one-week history of low back pain. Positron emission tomography–computed tomography (PET-CT) revealed hypermetabolic masses in the anterior mediastinum, adjacent to right cardiophrenic angle, in the retroperitoneum, and adjacent to the left iliac vessels in the pelvis. The puncture biopsy of the mass adjacent to the cardiophrenic angle suggested LELC coexisting with NHL-T. The patient was treated with a combination regimen of a PD-1 inhibitor and platinum-based chemotherapy, but disease progression was observed. Subsequent biopsy of the pelvic mass confirmed the presence of NHL-T. The treatment regimen was then modified to include a PD-1 inhibitor, bortezomib, and ICE (ifosfamide, carboplatin, etoposide) chemotherapy. After two cycles, the response was assessed as partial remission. The patient remains on ongoing therapy. This case highlights diagnostic challenges and provides insights into the management of such rare collision tumors.

## Introduction

Lymphoepithelioma-like carcinoma (LELC) is a rare malignant neoplasm characterized by distinct histopathological features, including nests of undifferentiated or poorly differentiated epithelial tumor cells extensively infiltrated by lymphocytes and plasma cells, forming a characteristic “lymphoepithelioma-like” architecture (1). T-cell non-Hodgkin lymphoma (NHL-T) constitutes a heterogeneous group of malignancies derived from mature T lymphocytes (2). Lymphomatous cells display variable morphology, ranging from small to large cells (3), with nuclei that may be round or irregularly shaped - such as convoluted, cerebriform, or lobulated - and exhibit considerable variation in cytoplasmic volume (4). When two histologically distinct tumors arise in close proximity within the same anatomical site and merge into a single mass, the entity is termed a collision tumor (5). Collision tumors comprising LELC and lymphoma are exceedingly rare, with only one prior report describing a combination of LELC and diffuse large B-cell lymphoma (6). To our knowledge, no previous cases of collision tumors involving LELC and T-cell lymphoma have been documented. Furthermore, due to the dense lymphocytic stroma inherent to LELC, which may mimic lymphoma, one component of the dual pathology may be overlooked, posing significant diagnostic challenges. Herein, we describe an exceptionally rare case of a collision tumor composed of LELC and NHL-T.

## Case presentation

**Figure 1:**
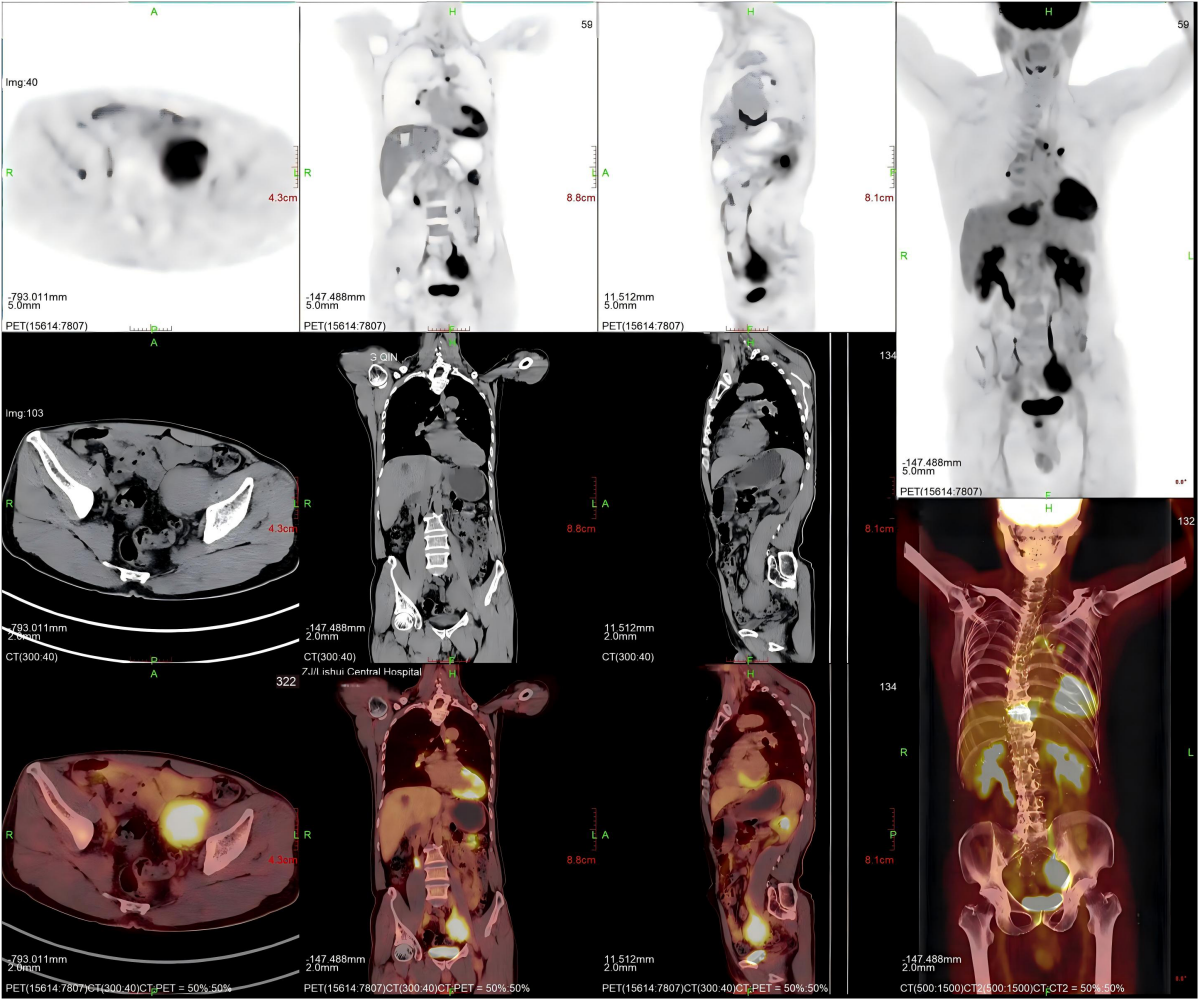
PET-CT revealed hypermetabolic masses in the anterior mediastinum, adjacent to right cardiophrenic angle, in the retroperitoneum, and adjacent to the left iliac vessels in the pelvis.

**Figure 2:**
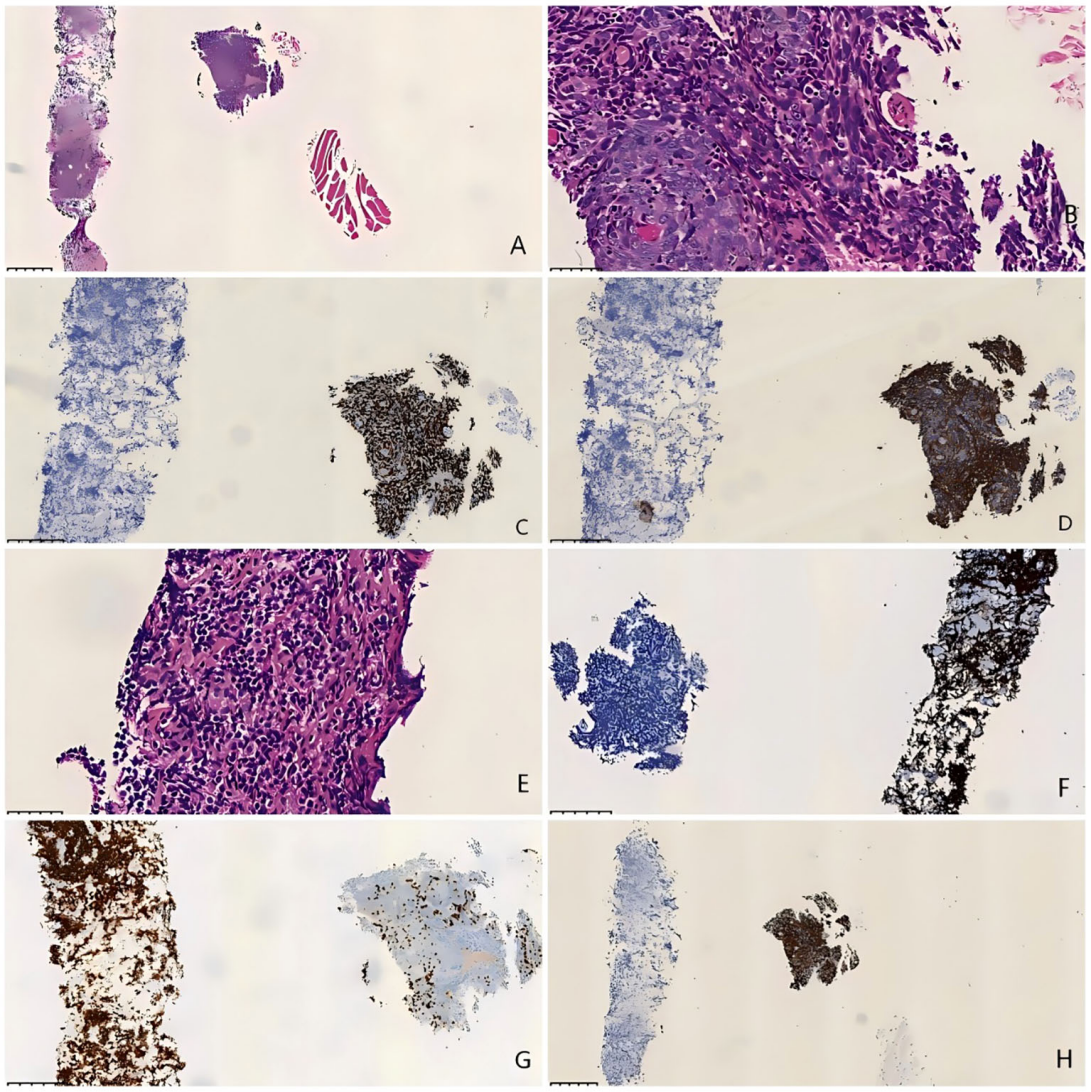
**(A)** Histopathological examination of the percutaneous biopsy specimen from the mass adjacent to the cardiophrenic angle: One component consisted of poorly differentiated carcinoma cells arranged in nests, with a lymphocytic background. The other component was composed of medium-sized lymphocytes growing in diffuse sheets.(hematoxylin and eosin, ×50). **(B)** The tumor cells exhibited a nested growth pattern, characterized by large, hyperchromatic nuclei and conspicuous nucleoli.(hematoxylin and eosin, ×400). **(C)** Immunohistochemical results showed that P40 was positive in lymphoepithelioma-like carcinoma cells and negative in the lymphoma component. (EnVision, ×100). 2 **(D)** Immunohistochemical results showed that AE1/AE3 was positive in lymphoepithelioma-like carcinoma cells and negative in the lymphoma component. (EnVision, ×100). **(E)** A prominent proliferation of atypical medium-sized lymphocytes was observed in a diffuse, sheet-like pattern, featuring fine chromatin and inconspicuous nucleoli.(EnVision, ×400) **(F)** Immunohistochemistry demonstrated CD56 positivity in non-Hodgkin T-cell lymphoma.(EnVision, ×100) **(G)** Immunohistochemistry demonstrated CD3 positivity in non-Hodgkin T-cell lymphoma.(EnVision, ×100). **(H)** Immunohistochemistry demonstrated EBER positivity in lymphoepithelioma-like carcinoma cells, but EBER negativity in non-Hodgkin T-cell lymphoma. (EnVision, ×50).

**Figure 3:**
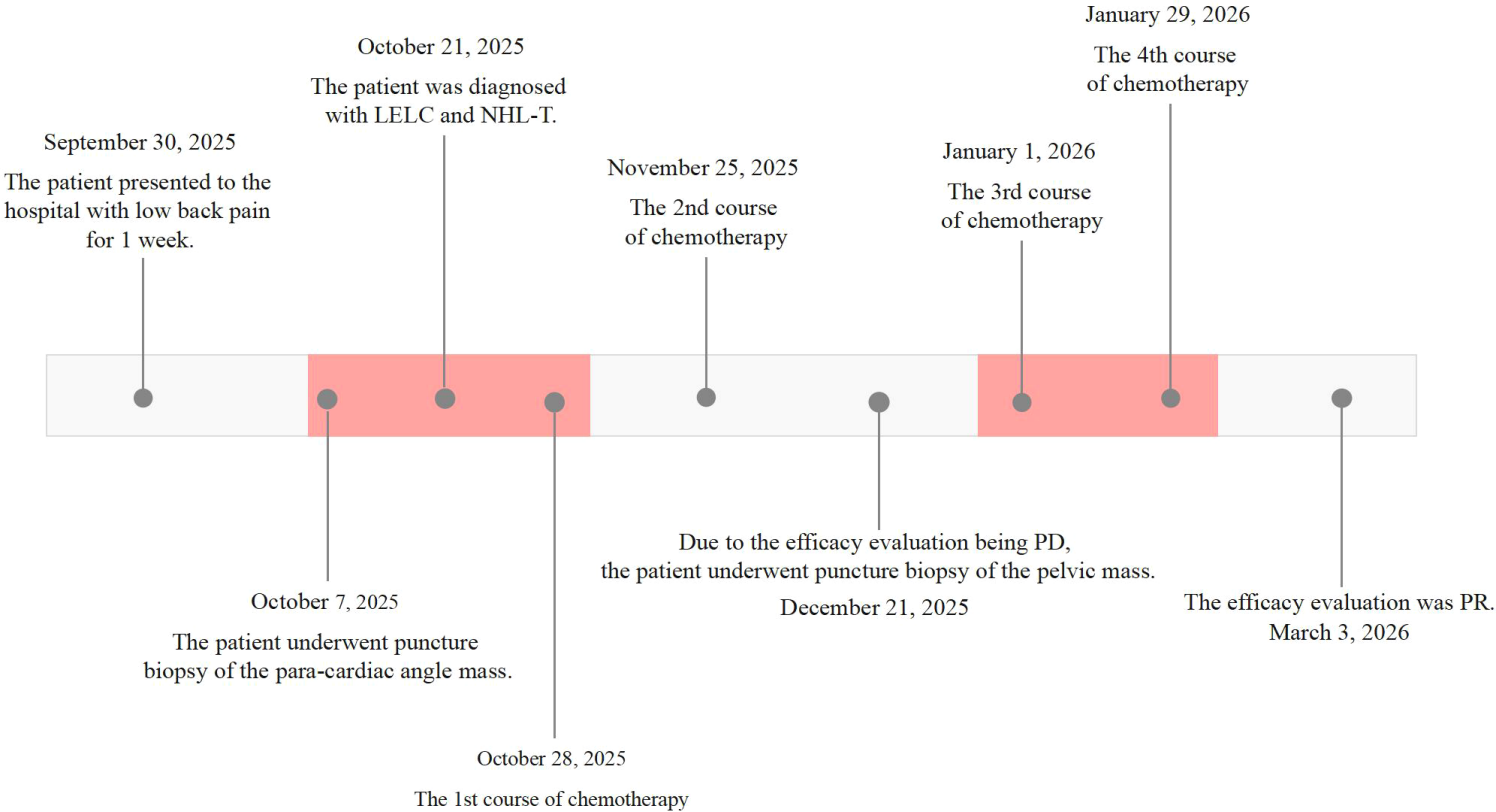
Clinical timeline of the patient.

The patient was a 61-year-old male with previous good health, no family history of malignancies and no history of smoking or excessive alcohol consumption. He presented to Lishui Central Hospital on September 30, 2025, complaining of low back pain persisting for one week. Physical examination revealed no palpable enlargement of superficial lymph nodes, no hepatosplenomegaly, and positive percussion tenderness over the left renal area. Laboratory evaluations including complete blood count, liver and renal function tests, and coagulation profile showed no significant abnormalities. Tumor markers (CEA, CA199, AFP, and SCC) were within normal ranges. Antinuclear antibody testing was negative; Epstein-Barr virus (EBV) DNA was undetectable, and EBV IgM and IgA antibodies were normal. Serologic tests for hepatitis B, hepatitis C, syphilis, and HIV were all negative. Lactate dehydrogenase level was normal. Positron emission tomography-computed tomography (PET-CT) revealed masses in the anterior mediastinum, adjacent to the right cardiophrenic angle, in the retroperitoneum, and adjacent to the left iliac vessels in the pelvic cavity; the pelvic mass involved the distal left ureter, resulting in secondary hydronephrosis of the left urinary system (**Figure 1**). These lesions exhibited increased glucose metabolism, with a maximum standardized uptake value of 7.5. The interface between the lesion and the adjacent ureter was indistinct, and dilatation with hydronephrosis was observed in the proximal ureter, left renal pelvis, and calyces. Subsequently, a percutaneous biopsy was performed on the mass near the right cardiophrenic angle. Histopathological analysis revealed two distinct neoplastic components (**Figure 2A**). One component comprised multifocally distributed nests of atypical epithelial cells displaying a syncytial growth pattern, set against a background of lymphocytic infiltration (**Figure 2B**). These epithelial cells displayed indistinct cell membranes, scant cytoplasm, vesicular nuclei, prominent nucleoli, and frequent mitotic figures. Immunohistochemical staining of these cells showed positivity for AE1/AE3, focal EMA, 34βE12, p40, and CD138; negativity for GATA3, CK7, CK20, CD3, CD20, CD30, CD56, TdT, and CD5. The Ki-67 proliferation index was 60%, p53 expression followed a wild-type pattern, and EBER *in situ* hybridization was positive (**Figures 2C, D**). Additionally, numerous atypical medium-sized lymphoid cells were identified, proliferating in diffuse sheets and infiltrating fibrous stroma. These lymphoid cells had fine chromatin and inconspicuous nucleoli (**Figure 2E**). Immunohistochemical analysis revealed expression of CD3, CD56, and GATA3; absence of reactivity for AE1/AE3, EMA, 34βE12, CK7, CK20, p40, CD5, CD20, CD30, CD138, and TdT. The Ki-67 index was 65%, p53 showed a wild-type pattern, and EBER *in situ* hybridization was negative (**Figures 2F–H**). Based on histomorphology and immunophenotypic findings, a diagnosis of LELC and NHL-T was established, fulfilling the criteria for a collision tumor. Next-generation sequencing of the tissue sample identified a nonsense mutation (p.K145*) and a splice-site mutation (c.4455-2A>T) in the *SETD2* gene, with variant allele frequencies of 29.8% and 30.0%, respectively. Bone marrow examination showed no evidence of tumor involvement. The patient was diagnosed with Lugano stage IV NHL-T and had an International Prognostic Index score of 3, accompanied by LELC with undetermined clinical stage. The patient subsequently received chemotherapy comprising a PD-1 inhibitor combined with platinum-based agents, detailed as follows: sintilimab 200 mg on day 1; cyclophosphamide 650 mg/m² on day 2; liposomal doxorubicin 25 mg/m² on day 2; dexamethasone 15 mg daily from day 2 to day 6; cisplatin 30 mg/m² on day 4. All agents were administered by intravenous infusion, with cycles repeated every 28 days. After two cycles, follow-up CT imaging showed reduction in the size of the cardiophrenic angle mass from 59 mm × 31 mm to 38 mm × 16 mm. However, the pelvic mass increased from 49 mm × 45 mm to 61 mm × 60 mm, indicating progressive disease. We then performed a puncture biopsy of the pelvic mass, and the pathology indicated NHL-T. The treatment regimen was subsequently adjusted to a combination of a PD-1 inhibitor, bortezomib, and the ICE regimen, with the specific dosage schedule as follows: sintilimab 200 mg on day 1; ifosfamide 2 g/m² on days 2-3; carboplatin at an AUC of 5 on day 2; etoposide 75 mg/m² on days 1-3; bortezomib 1.3 mg/m² on days 1, 4, 8 and 11. All agents were administered by intravenous infusion except bortezomib, which was given via subcutaneous injection, with treatment cycles repeated every 28 days. Following two cycles of the revised regimen, repeat CT demonstrated further shrinkage of the cardiophrenic angle mass to 17 mm × 9 mm and reduction of the pelvic mass to 35 mm × 26 mm, consistent with partial response. During treatment, the patient developed grade IV myelosuppression accompanied by infectious fever, which resolved following aggressive symptomatic and supportive management. The patient remains on a continuous therapeutic regimen. The overall follow-up period spanned six months from initial pathological diagnosis to the latest evaluation. The patient reported favorable treatment tolerability, with a mild decline in overall quality of life. The patient’s clinical timeline is shown in **Figure 3**.

## Discussion

A collision tumor refers to the simultaneous presence of two or more histologically distinct malignant neoplasms in the same organ or anatomical site (7). Both LELC and T-cell lymphoma are rare entities, and their co-occurrence as a collision tumor is exceedingly uncommon. To date, no such cases have been reported in the literature.

LELC can occur at multiple sites throughout the body, with the most common locations being the lungs, stomach, salivary glands, and thymus (8). Its pathological features include nests of syncytial-growing epithelial tumor cells infiltrated by numerous reactive lymphocytes and plasma cells, forming a distinctive “lymphoepithelial” pattern (1). Accumulating evidence indicates that EBV infection is closely linked to the pathogenesis of LELC, with EBV positivity rates exceeding 90% in LELC cases originating in the nasopharynx, stomach, and lungs (9). The clinical presentation of LELC is nonspecific and largely depends on the primary tumor site. Patients with pulmonary LELC are often asymptomatic and are typically diagnosed incidentally during routine physical examinations or imaging studies performed for unrelated indications, presenting radiologically as solitary pulmonary nodules or masses (10) (11). Gastric LELC may manifest endoscopically as ulcers or discrete masses, with patients frequently seeking medical evaluation due to symptoms such as abdominal pain, dyspepsia, or gastrointestinal bleeding (12). NHL-T represents a heterogeneous group of malignant neoplasms derived from mature T lymphocytes, characterized by aggressive clinical behavior and suboptimal responses to conventional chemotherapy regimen (13). It encompasses several subtypes, including peripheral T-cell lymphoma, angioimmunoblastic T-cell lymphoma, anaplastic large-cell lymphoma, and NK/T-cell lymphoma (2). Clinical manifestations of NHL-T vary according to subtype but commonly include painless progressive lymphadenopathy, fever, night sweats, and unintentional weight loss - so-called B symptoms (14). In this case, the patient presented with low back pain. Imaging revealed multifocal tumor involvement, including the anterior mediastinum, the right cardiophrenic angle region, the retroperitoneum, and the pelvis. Histopathological examination of a core needle biopsy from the mass adjacent to the cardiophrenic angle demonstrated two distinct neoplastic components under microscopic evaluation. One component consisted of poorly differentiated carcinoma cells arranged in nests, with a lymphocytic background. These tumor cells displayed a syncytial growth pattern, indistinct cell borders, scant cytoplasm, vesicular nuclei, prominent nucleoli, and frequent mitotic figures. The second component was composed of medium-sized lymphoid cells proliferating in diffuse sheets, with fine chromatin and inconspicuous nucleoli. Immunohistochemical analysis of the carcinoma component showed positive staining for AE1/AE3 and p40, confirming epithelial differentiation. Furthermore, EBER *in situ* hybridization was positive, supporting a diagnosis of EBV-associated LELC. In contrast, the lymphoid component expressed CD3 and CD56, consistent with a T-cell lineage, thereby confirming the diagnosis of T-cell lymphoma. However, the pathological tissue specimens of this patient were obtained via deep mass puncture, and the available tissue volume was too limited to allow further supplementary immunohistochemical staining for definite subtyping of NHL-T. Notably, EBER *in situ* hybridization was negative in this lymphoid population, indicating absence of EBV infection in the lymphomatous cells-an observation distinct from the EBV-positive carcinoma component. Based on cytomorphological features, immunophenotypic profiles, and differential EBER expression, a composite diagnosis of coexisting LELC and NHL-T was established. Importantly, in this case, the lymphocytic infiltration surrounding the LELC represented a classic histological feature of this carcinoma. However, it must be emphasized that such lymphocytes are not invariably reactive. The high proliferation indices of CD56 and Ki-67 observed in the lymphoid component supported its neoplastic nature, indicating clonal T-cell lymphoma rather than reactive lymphocytic hyperplasia. Therefore, in the setting of prominent lymphoid infiltration, pathologists must carefully assess spatial architecture, cytological morphology, immunohistochemical markers, and molecular findings to avoid overlooking an accompanying hematopoietic malignancy. A subsequent pathological evaluation (core needle biopsy of the pelvic mass) confirmed the presence of NHL-T alone. Thus, it was concluded that the lesion at the cardiophrenic angle represented a collision tumor comprising both LELC and T-cell lymphoma, whereas the pelvic mass consisted predominantly of T-cell lymphoma.

The pathogenesis of collision tumors remains unclear, with several hypotheses proposed to date. The most widely accepted is the polyclonal origin theory, which posits that collision tumors arise from two or more independent progenitor or precursor cell clones. Under the influence of the same or distinct carcinogenic factors, these clones undergo independent malignant transformation and proliferation, ultimately converging anatomically to form a single mass (15). Molecular analyses of different tumor components in collision tumors provide supportive evidence for this hypothesis, often revealing distinct mutational profiles. For example, in a collision tumor composed of diffuse large B-cell lymphoma and colon adenocarcinoma, the lymphoma component harbored a *TP53* mutation, whereas the adenocarcinoma component exhibited a *BRAF* mutation and *MLH1* promoter methylation (16). In the present case, next-generation sequencing was performed on the two tumor components. No disease-associated gene mutations were detected in the LELC component, while the lymphoma component harbored a nonsense mutation (p.K145*) and a splice site mutation (c.4455-2A>T) in the *SETD2* gene. *SETD2* is a critical epigenetic regulatory gene, encoding the sole histone methyltransferase responsible for trimethylation of lysine 36 on histone H3 (H3K36me3) (17). Previous studies have reported *SETD2* mutations in mature T/NK-cell lymphomas, including enteropathy-associated T-cell lymphoma and hepatosplenic T-cell lymphoma (18) (19), suggesting a potential pathogenic role in T-cell lymphomagenesis. Although *SETD2* mutations have also been identified in certain solid tumors, such as renal cell carcinoma, glioma, and breast cancer, no established association exists between *SETD2* mutations and LELC. Therefore, it is inferred that the T-cell lymphoma and LELC components in this collision tumor originated from distinct clonal lineages. Another hypothesis involves the tumor microenvironment. Shared microenvironmental insults, including chronic inflammation and specific pathogen infections, can concurrently induce and promote malignant transformation of distinct cell populations within the same anatomical region (20). EBV infection is closely associated with the pathogenesis of LELC (9). In the present case, EBV expression was detected in the LELC component but absent in the lymphoma counterpart. This finding indicates that EBV is not implicated in lymphomagenesis herein, suggesting that the coexistence of the two neoplasms in this patient is likely an incidental collision event.

Currently, there is no standardized treatment approach for collision tumors; management decisions must be individualized based on the constituent tumor types. For locally resectable cases, radical surgery remains the primary therapeutic modality, with postoperative adjuvant therapy tailored according to the biological behavior of the dominant or more aggressive component (21). In patients with advanced, unresectable disease, treatment strategies should aim to encompass the characteristics of all tumor components as comprehensively as possible (22). For early-stage, resectable LELC, radical surgical resection is the treatment of choice (10). However, no uniform protocol exists for advanced-stage LELC, where platinum-based combination chemotherapy regimens are currently considered first-line therapy in clinical practice (23). Moreover, accumulating evidence supports the efficacy of PD-1 inhibitors in advanced LELC (24) (25). Conventional chemotherapeutic regimens, such as CHOP, demonstrate limited effectiveness in treating T-cell lymphoma (26). Recently, targeted therapies including brentuximab vedotin and histone deacetylase inhibitors, along with immunotherapies such as immune checkpoint inhibitors and novel bispecific antibodies, have improved outcomes in specific T-cell lymphoma subtypes (27) (28) (29). In this case, involving an unresectable collision tumor of LELC and T-cell lymphoma with extensive disease burden, initial systemic therapy with a PD-1 inhibitor combined with a platinum-based regimen yielded suboptimal response. Numerous studies have demonstrated that bortezomib is effective and well-tolerated in the management of T-cell lymphoma (30, 31), therefore, a proteasome inhibitor was incorporated into the regimen, and treatment was switched to PD-1 inhibitor plus bortezomib in combination with ICE (ifosfamide, carboplatin, etoposide), resulting in partial remission. The patient is currently undergoing continued cycles of chemotherapy.

This case report has several limitations that should be acknowledged. First, the follow-up period was relatively short, which limits the evaluation of long-term therapeutic efficacy and late adverse events. Secondly, limited punctured tissue material precluded TCR gene rearrangement analysis and further subtyping of NHL-T. Thirdly, digestive endoscopy or nasopharyngoscopy was not performed to identify the primary origin of LELC. Finally, as a single-case report, this study bears inherent limitations, including poor generalizability and the inability to draw definitive conclusions.

## Conclusion

In conclusion, we present a rare case of a collision tumor comprising LELC and NHL-T in a patient presenting with low back pain. The histological subtypes were confirmed by pathological examination, and the underlying pathogenesis was further explored through molecular testing. This case offers valuable insights for the diagnosis and management of similar entities in clinical practice.

## Data Availability

The original contributions presented in the study are included in the article/supplementary material. Further inquiries can be directed to the corresponding author.
